# Advances in Carbon Xerogels: Structural Optimization for Enhanced EDLC Performance

**DOI:** 10.3390/gels10060400

**Published:** 2024-06-14

**Authors:** Jongyun Choi, Ji Chul Jung, Wonjong Jung

**Affiliations:** 1 Department of Chemical Engineering, Myongji University, Yongin 17058, Republic of Korea; jongyun0122@gmail.com; 2 Department of Mechanical, Smart, and Industrial Engineering, Gachon University, Seongnam 13120, Republic of Korea

**Keywords:** carbon xerogels, tailoring structures, EDLCs, electrochemical performance

## Abstract

This review explores the recent progress on carbon xerogels (CXs) and highlights their development and use as efficient electrodes in organic electric double-layer capacitors (EDLCs). In addition, this work examines how the adjustment of synthesis parameters, such as pH, polymerization duration, and the reactant-to-catalyst ratio, crucially affects the structure and electrochemical properties of xerogels. The adaptability of xerogels in terms of modification of their porosity and structure plays a vital role in the improvement of EDLC applications as it directly influences the interaction between electrolyte ions and the electrode surface, which is a key factor in determining EDLC performance. The review further discusses the substantial effects of chemical activation with KOH on the improvement of the porous structure and specific surface area, which leads to notable electrochemical enhancements. This structural control facilitates improvement in ion transport and storage, which are essential for efficient EDLC charge–discharge (C–D) cycles. Compared with commercial activated carbons for EDLC electrodes, CXs attract interest for their superior surface area, lower electrical resistance, and stable performance across diverse C–D rates, which underscore their promising potential in EDLC applications. This in-depth review not only summarizes the advancements in CX research but also highlights their potential to expand and improve EDLC applications and demonstrate the critical role of their tunable porosity and structure in the evolution of next-generation energy storage systems.

## 1. Introduction

With the growing interest in the development and utilization of eco-friendly energy sources, the research on electrical energy storage devices is becoming increasingly active with the goal of ensuring the efficient use of electrical energy [1,2,3,4,5,6,7]. Such energy storage devices include fuel cells, lithium-ion batteries (LIBs), and electric double-layer capacitors (EDLCs), with each device finding its utilization in various fields depending on its unique advantages [8,9,10,11,12]. EDLCs are extensively used in industries such as electric vehicles, portable electronic devices, and renewable energy systems, owing to their rapid charge–discharge (C–D) capability, excellent durable life cycles, and high power density [13,14,15,16]. The electrode materials in EDLCs consist of carbon material as the active material, a conductive additive, and a polymer binder. Active materials, including activated carbons, carbon nanotubes, activated carbon fibers, and carbon xerogels (CXs), considerably influence the performance of EDLCs [17,18,19,20,21,22,23,24,25,26]. Specifically, due to their chemical stability, cost-effectiveness, porous structure, and high specific surface area, activated carbons are commercially preferred as the active materials for EDLCs [17,27]. However, the low electrical conductivity of activated carbons limits their use as electrode materials [27,28]. Therefore, many studies should focus on active materials superior to activated carbons for applications in EDLC electrodes.

Our research group concentrated on CXs, which, although they have similar advantages to activated carbon, possess outstanding electrical conductivity. CXs exhibit remarkable electrical conductivity due to their effective connectivity via three-dimensional (3D) networks of carbon particles [29,30,31]. Furthermore, physical properties, such as 3D network structure, pore size, and specific surface area of CXs, can be systematically controlled through the adjustment of synthesis parameters, which makes them highly promising as electrode materials for EDLCs [32,33,34,35]. For these reasons, numerous studies have investigated the mechanism underlying the electrochemical performances of EDLCs under the influence of various pore sizes of CXs, which occur due to synthesis conditions and the physical characteristics of CXs [36,37,38,39,40,41,42,43,44]. In addition, various attempts, including methods beyond structure control, such as metal doping and the application of activation processes to CXs, are being made to improve the performance of EDLC electrodes through CXs [37,43,44,45,46].

**Figure 1:**
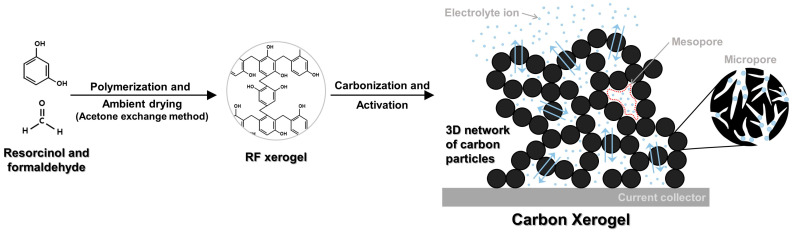
Schematic summary of this review.

This review comprehensively introduces the methodologies used for the fabrication of CXs, with a focus on the structural control of CXs to specialize them for application in EDLC electrodes. This work further delves into CX electrochemical characteristics and their potential for industrial application in EDLC electrodes, various approaches utilized for the synthesis of CXs as electrode materials for EDLCs, and the effects of synthesis parameters on the electrochemical performance of EDLCs [47,48,49,50,51,52,53,54,55,56,57,58,59,60,61]. Specifically, an analysis was conducted on the effects of synthesis parameters, such as the polymerization duration, pH, and amount of catalyst on the physical properties and electrochemical performance of CXs [55,56,57]. Furthermore, a comparison with commercial electrode materials for EDLC electrodes was conducted to explore the commercial viability of CXs [58]. In our studies, we used an organic electrolyte and a two-electrode system, which mirror the workings of commercial EDLCs, and the results imply the great industrial relevance of our findings. This review is of considerable importance, as it compares and analyzes data collected over more than a decade of research conducted by a single research group under nearly identical conditions. This allows for a more consistent explanation of the effects of varying experimental parameters. Figure 1 provides a summary of this review.

## 2. CXs

**Figure 2:**
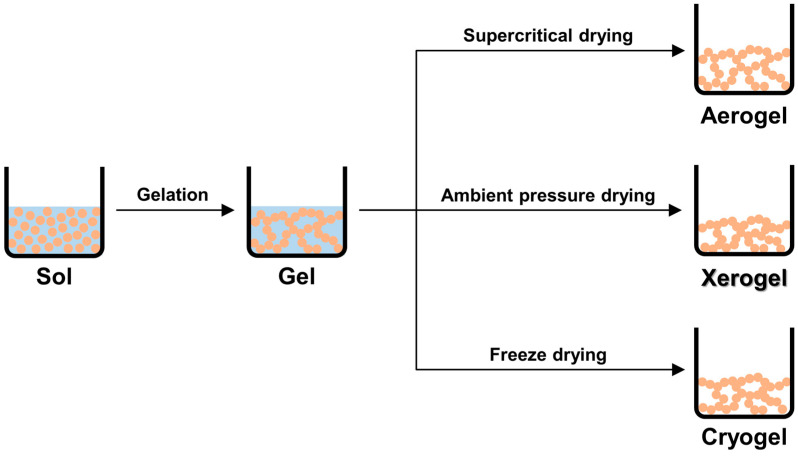
Synthesis processes of various carbon gels [63,64].

CXs represent a type of carbon gel obtained through the carbonization of organic gels. In addition to xerogels, carbon gels include aerogels and cryogels, which are differentiated through the drying method of wetted organic gels [62,63,64]. Figure 2 illustrates the synthesis processes of various carbon gels, including carbon aerogels, xerogels, and cryogels, and Table 1 summarizes their characteristics. Aerogels can be obtained through supercritical drying to prevent the collapse of their 3D network structures caused by the strong capillary forces of water [65]. Cryogels are synthesized via freeze drying to facilitate the removal of water from the gels [66]. Notably, xerogels can be easily prepared through drying under ambient temperature and pressure conditions and can be produced by exchanging the water in the hydrogels with a substance of low surface tension before drying to prevent the collapse of their 3D network structures [62,67]. Xerogels with a stable pore structure were successfully synthesized by replacing the water in hydrogels with acetone, which has a lower surface tension, followed by drying [47,68,69]. The advantages of xerogels include a simple production method and the lack of a need for complex equipment, which makes them suitable for mass production [68]. Therefore, xerogels have a higher commercial potential compared with other carbon gels.

CXs are typically synthesized via the sol–gel method, which involves the use of resorcinol and formaldehyde as reactants and sodium carbonate (Na_2_CO_3_) as a base catalyst to produce a resorcinol–formaldehyde (RF) gel [67,69]. The physical properties of CXs can be adjusted based on the reaction conditions during RF gel formation [55,56,57,69,70,71]. The general procedure for CX fabrication includes the following steps: (1) initiation of polymerization through the addition of resorcinol and formaldehyde to an aqueous solution containing the base catalyst Na_2_CO_3_, which results in sol formation; (2) aging and gelation, which leads to the formation of hydrogels with a 3D network structure; (3) implementation of the acetone exchange method, followed by drying under ambient pressure and temperature conditions to produce RF xerogels; and (4) carbonization of RF xerogels under an inert atmosphere to obtain CXs.

The physical properties of CXs, such as specific surface area, pore size, pore volume, and 3D network structure, are determined by their synthesis parameters, including polymerization duration, pH, and the ratio of resorcinol to catalyst (R/C ratio). CXs with tailored structures and physical properties for specific applications can be fabricated through precise control of the synthesis parameters [72,73,74]. Therefore, CXs have attracted considerable interest for various applications. For example, CXs are utilized in biomedical applications due to the porosity obtained from the sol–gel process. CXs can be engineered to be texturally suited for specific drugs, thereby optimizing effective drug delivery [62,75]. Moreover, the inherent chemical stability of carbon materials and the adjustable porous properties of CXs have been employed as supports for metal catalysts such as Pt/C and Ru/C [76]. This is particularly useful in advanced oxidation processes, such as wet air oxidation, and in producing fine chemicals. Furthermore, CXs are utilized in environmental engineering. Discharging industrial effluents containing toxic metal ions and persistent reactive dyes into the environment presents significant life-threatening effects, highlighting the urgent need for efficient solutions. CXs have shown effectiveness in adsorbing large organic molecules from wastewater, and their commercial applications are under exploration [77,78,79].

Most importantly, CXs show great potential uses in energy storage devices, such as fuel cells, LIBs, and EDLCs [80,81,82]. Research on the use of CXs in energy storage devices draws significant interest because the structural control of CXs can substantially improve the performance of these devices [81,83]. In particular, the use of CXs is gaining attention in the field of EDLCs, where the structural and physical properties of active materials considerably influence the electrochemical performance [84,85]. Their adjustable physical properties based on reaction conditions suggest the high utility of CXs as electrode materials for EDLCs. Therefore, we synthesized structurally controlled CXs by regulating the reaction conditions and used them as active materials for EDLC electrodes [52,53,54,55,56,57,58]. In addition, we conducted studies to understand the interrelation between the physical properties of CXs, determined by the synthesis parameters, and the electrochemical performance of EDLCs.

## 3. EDLC

EDLCs, known as supercapacitors, derive their name from their working principle. EDLCs store energy through electrolyte ion adsorption on the electrode surface, which forms a Helmholtz double layer and utilizes electrostatic attraction [86,87]. Importantly, EDLCs store electrical energy via the adsorption–desorption process of electrolyte ions on the electrode surface. Based on this operation principle, EDLCs offer several advantages, including long-term durability, rapid C–D rates, and high power densities [88]. In contrast, LIBs store electrical energy through redox reactions at the electrodes. Specifically, compared to LIBs, which maintain 80% of their initial capacity after 3000 cycles, EDLCs exhibit significantly long-term durability, maintaining over 80% of their capacity even after 10,000 cycles [89,90,91]. This difference makes EDLCs and LIBs complementary to each other, as both types of devices bring unique benefits and offset each other’s limitations [88]. These complementary characteristics enable the combined application of both types of energy storage devices across various industries [92].

Combining EDLCs with LIBs creates a powerful energy management system capable of handling sudden increases in electricity demand. This effectiveness is due to the ability of EDLCs to charge and discharge rapidly. For instance, EDLCs help stabilize the power grid by instantly supplying power, which is particularly useful for balancing the unpredictable nature of renewable energy sources like solar and wind [13]. Additionally, EDLCs significantly enhance the efficiency and overall performance of electric vehicles and hybrid electric vehicles by improving energy use during crucial actions such as regenerative braking and acceleration [16]. Moreover, by adeptly managing high-power demands, EDLCs help extend the lifespan of these vehicles’ batteries. Consequently, EDLCs make substantial contributions to various fields, including renewable energy systems, uninterruptible power supplies, and electric vehicles [93,94]. The pivotal role of energy storage technologies, particularly EDLCs, in balancing energy supply and demand and enhancing the reliability of the electricity supply cannot be overstated.

The electrochemical performance of EDLCs is considerably affected by several key components, including the electrolyte, conducting additive, binder, and active material [95,96,97,98,99,100,101]. Electrolytes can be divided into aqueous and organic types, with the latter being preferred in the industry due to their higher electrical conductivity and stability at elevated voltages [102,103]. Conducting additives, such as carbon black, increase the electrical conductivity of the electrodes [98]. Binders, such as polytetrafluoroethylene and polyvinylidene fluoride (PVDF), bond the conductive additive and active material to the current collector [99]. The active material, which provides the necessary space for electrolyte ion adsorption, greatly influences the EDLC performance [100,101]. To enhance the performance of EDLCs, researchers have utilized carbon materials, such as activated carbons, carbon nanotubes, activated carbon fibers, and CXs, with extensive studies being conducted on active materials [101,104,105]. Table 2 provides a summary of the results of relevant studies that employed a two-electrode system analogous to commercial EDLCs.

The physical properties of electrode materials, including their specific surface areas, pore sizes, and pore volumes, are pivotal determinants of EDLC performance [97,98]. This highlights the importance of understanding the relationship between the physical properties of electrode materials and the electrochemical performance of EDLCs. Notably, EDLCs exhibit a markedly improved electrochemical performance when their pore size closely matches that of the electrolyte ions [135,136]. In addition, a high specific surface area is crucial for the enhanced performance of EDLCs.

According to the International Union of Pure and Applied Chemistry (IUPAC) standards, electrode material pores can be categorized based on their diameters into micropores (widths not exceeding approximately 2 nm), mesopores (between 2 and 50 nm), and macropores (exceeding 50 nm) [137]. Among these pores, the micropores and mesopores within active materials considerably affect EDLC performance. Electrolyte ion adsorption is highly influenced by the specific surface area of active materials, which is largely determined by the volume of micropores and mesopores. In addition, mesopores act as conduits for ion movement, which affects the electrochemical performance of EDLCs [138]. However, EDLC electrochemical performance is not entirely dependent on the specific surface area or pore size [139]. Various factors, such as electrical conductivity and the composition of the 3D network structure, exert a complex influence [88]. Consequently, research and development should focus on active materials for EDLCs with precisely controlled properties, including electrical conductivity, pore structure, and specific surface area.

CXs can have a tailored pore volume and size through the adjustment of synthesis parameters. Moreover, the 3D carbon particle network of CXs can improve electrical conductivity. This property has prompted numerous researchers to investigate CXs as EDLC electrode materials. To enhance EDLC performance with CXs, researchers have proposed strategies that not only control physical properties but also include metal doping [45,46,140]. Doping CXs with metals such as Co, Ni, Cu, Mn, Fe, and Zn considerably increases the specific capacitance of EDLCs, and such an improvement is attributed to the presence of metal oxides and the resulting Faradaic redox reactions [48,49,50,51]. However, redox reactions can reduce some key benefits of EDLCs, including durable life cycles and power density. Therefore, optimization of the CX structure has been found to be more effective than metal doping for achieving high-performance EDLCs.

**Figure 3:**
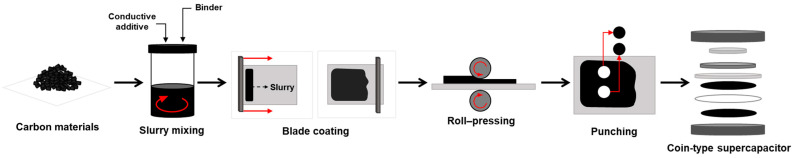
Scheme of the coin-cell EDLC fabrication process [22,111,115].

Coin-type EDLCs were assembled to analyze the electrochemical performance of CXs, with the fabrication process detailed in Figure 3. The electrodes were produced using various carbon materials (CXs, activated CXs (ACXs), and activated carbons) as the active material, Super-P as the conducting additive, and PVDF as the binder. A slurry was prepared by mixing the active material, conductive agent, and binder at a mass ratio of 8:1:1 with 1-methyl-2-pyrrolidone as the solvent. Then, the slurry was coated onto an Al current collector. The organic electrolyte 1 M tetraethylammonium tetrafluoroborate dissolved in acetonitrile (1 M TEABF_4_/ACN) was utilized in the coin-type EDLCs. The assembled EDLCs were evaluated for their electrochemical performance through cyclic voltammetry (CV), galvanostatic charge–discharge (GCD), electrochemical impedance spectroscopy (EIS), and four-point probe (FPP) measurements.

## 4. Tailoring Structure of Carbon Xerogels by Synthesis Parameters

**Figure 4:**
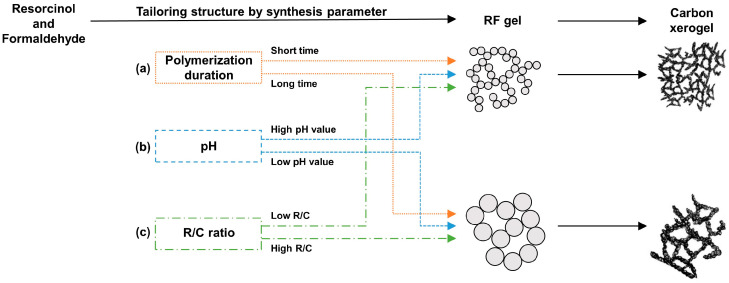
Structural changes of CXs based on synthesis parameters: (**a**) polymerization duration, (**b**) pH, and (**c**) R/C ratio [55,56,57,142,143].

Xerogels were produced by subjecting RF gels to the drying–wetting method under ambient conditions. During the drying–wetting process, the high surface tension of water (71.99 dyne/cm) posed a risk of pore structure collapse [141]. To mitigate this issue, our research group replaced the water in the RF gels with acetone, which has a lower surface tension (20.66 dyne/cm), before proceeding to the drying process [47]. As a result, the integrity of the pore structure was successfully preserved, which enabled the production of CXs with minimal structural collapse. The produced CXs were then utilized in our studies, which focused on designing CXs with precisely controlled structures through the manipulation of synthesis parameters. The synthesis process involved adjusting variables, including the polymerization duration, pH, and R/C ratio. Figure 4 concisely illustrates the structural alterations resulting from these varied conditions.

### 4.1. Polymerization Duration

As the physical properties of CXs varied depending on the RF gels prior to carbonization, understanding of the synthesis process of RF gels was essential. We optimized the polymerization duration to control the physical properties of the CXs, which led to an in-depth understanding of the RF gels’ synthesis process [55]. Adjusting the polymerization duration substantially affected the physical properties of the CXs, including their pore structure and specific surface area, which are vital for the electrochemical performance of EDLCs. Therefore, a systematic analysis is needed to determine the mechanism underlying the influence of polymerization duration on the physical properties of CXs and its consequent effects on the electrochemical performance of EDLCs. Figure 4a illustrates the variations in the CX structure over polymerization durations ranging from 1 h to 20 h.

**Figure 5:**
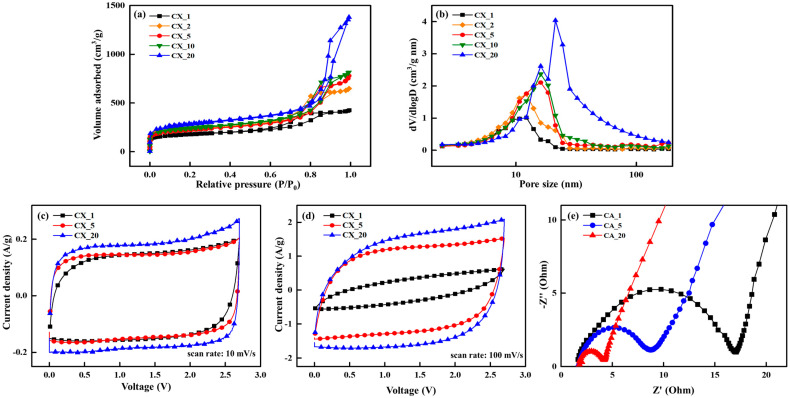
(**a**) N_2_ adsorption–desorption isotherm, (**b**) pore size distribution, (**c**) cyclic voltammograms at a scan rate of 10 mV/s, (**d**) cyclic voltammograms at a scan rate of 100 mV/s, and (**e**) Nyquist plots of carbon xerogels (CX_polymerization druation) [55].

Mesopore formation in CXs was evident across all polymerization durations (Figure 5a,b). The presence of these mesopores suggests the formation of a 3D network structure within CXs. Furthermore, the data presented in Table 3 show that the specific surface area, pore size, and pore volume increased with prolonged polymerization. This information is crucial for understanding the mechanism underlying the influence of polymerization duration on the structural characteristics of CXs. As the polymerization time increases, larger particle sizes could be generated [142]. As a result, these larger particles caused the creation of large pores and high pore volumes during the gelation process. Conversely, a short polymerization duration resulted in small molecular weights per particle, which led to the formation of smaller particles. These smaller particles formed a tightly interconnected 3D network structure within the CXs, which was characterized by small pore sizes and volumes. Therefore, the adjustment of polymerization duration plays a key role in the customizing of the structure of CXs, which makes it a critical parameter in the synthesis of CXs optimized for EDLC electrode materials.

CV and EIS analyses were conducted on the CXs (CX_1, CX_5, and CX_20), and the CV curves and Nyquist plots are illustrated in Figure 5c–e. Under optimal operational conditions, EDLCs maintain the magnitude of the current and linearly change in response to variations in voltage, irrespective of the flow direction [144]. Thus, an ideal CV curve should have a shape closely resembling a symmetrical rectangle [145]. At low scan rates under all polymerization durations, the CV curve maintained its rectangular shape. However, at a high scan rate of 100 mV/s, the shape of the CV curve transitioned from rectangular to being rugby-ball-shaped. This transformation indicates a decline in the electrochemical performance of the CXs. Notably, CX_1, which was synthesized within a short polymerization period, exhibited a more distorted rugby-ball-shaped CV curve at higher scan rates compared with other CXs. This condition prevented CX_1 from maintaining its capacitance at fast scan rates. This result can be attributed to the restricted mobility of electrolyte ions, as supported by the Nyquist plots obtained from EIS analysis.

The Nyquist plots were divided into three distinct sections: the bulk-solution resistance, which results from the mobility of electrolyte ions outside the electrode; the charge transfer resistance (R_ct_), which includes the inherent electronic resistance of the electrode material and the resistance against ion movement within it; and Warburg resistance, which arises from the diffusion of electrolyte ions and the internal resistance within the cell [23,24,25,26]. The semicircles in the Nyquist plots correspond to the R_ct_ at the electrode, with small semicircle diameters signifying a low R_ct_ [146]. Thus, the R_ct_ contributes to determining the intrinsic resistance of EDLC electrode materials.

CX_1 exhibited the lowest capacitance retention property and the highest R_ct_. Notably, the R_ct_ decreased with the extended polymerization duration (Figure 5e). This change in R_ct_ is closely linked to the structural transformations in CXs. Prolonged polymerization led to the formation of large particles, which in turn led to CXs with abundant pores. These pores facilitated electrolyte ion movement and acted as pathways for improved ion mobility within the CXs. As a result, enhanced electrolyte ion mobility was observed, reducing ion resistance. This outcome is consistent with the results obtained from other electrochemical analyses.

Thus, the control over the polymerization duration was a critical factor in the customization of CXs as active materials for EDLCs. The duration of polymerization profoundly influenced the growth of polymer particles. Notably, a short polymerization duration led to the formation of small particles. These small particles faced challenges in the formation of CXs with well-developed pores. The low porosity of CXs hindered the mobility of electrolyte ions, resulting in an increased R_ct_, which negatively influenced the overall electrochemical performance of the EDLCs. Therefore, methodical control of the polymerization time is crucial for the structural modification of CXs to optimize their effectiveness as electrode materials for EDLCs.

### 4.2. pH of Polymerization Solution

**Figure 6:**
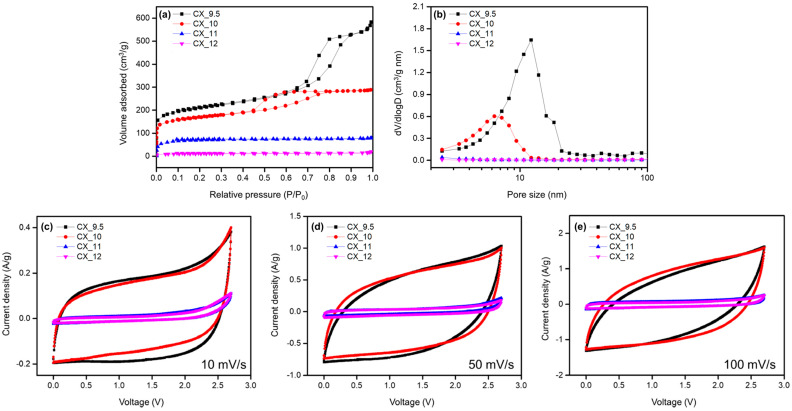
(**a**) N_2_ adsorption–desorption isotherm, (**b**) pore size distribution, and (**c**) cyclic voltammograms at a scan rate of 10 mV/s, (**d**) 50 mV/s, and (**e**) 100 mV/s of carbon xerogels (CX_pH) [56].

Figure 4b demonstrates the structural modifications that occurred in response to pH levels during the synthesis process of RF gels. Previous research has shown that pH levels significantly influence the formation of initial particle nuclei [142,146,147]. At high pH levels, numerous nuclei formed at the reaction initiation, leading to the formation of small particles due to spatial constraints and limited reactants. Through gelation, the small particles synthesized at high pH levels amalgamated to form a dense structure, which hindered pore formation. As indicated in Table 4 and Figure 6a,b, the CXs synthesized under high pH levels presented poorly developed pore structures and low specific surface areas. By contrast, synthesis at low pH levels resulted in the formation of fewer nuclei, which allowed for the growth of relatively large particles. These larger particles combined and formed a 3D network structure, resulting in the production of CXs with well-developed pores and high specific surface areas.

The electrochemical performance of EDLCs was substantially influenced by the specific surface area and structural characteristics of the active materials. For the CXs synthesized at relatively low pH levels (9.5 and 10), rectangular-shaped CV curves with a wide area were observed at various scan rates (Figure 6c–e). Although the geometry of the CV curve transformed from a rectangular to a rugby-ball shape with the increase in scan rates, the exceptional electrical conductivity of the 3D network structure ensured the preservation of a relatively rectangular shape. In summary, the CXs synthesized at low pH levels showed superior electrochemical performances, as evidenced by the CV curves.

Comparable outcomes were elucidated through GCD analysis. The specific capacitance (*C_g_*) derived from the GCD analysis was calculated using Equation (1), with the results tabulated in Table 4.

(1) $$C_{g}=\frac{I\cdot \Delta t}{m\cdot \Delta V}$$

where *C_g_* denotes the specific capacitance, m represents the mass of the active electrode material, Δ*V* indicates the voltage variation during the discharge phase, *I* is the current observed during discharge, and Δ*t* signifies the discharge duration.

CXs synthesized at low pH levels demonstrated superior specific capacitance across all current densities. These CXs possessed high specific surface areas, which contributed to their elevated specific capacitances at low C–D rates (low current density). Furthermore, the electrical conductivity facilitated by the 3D network connections and pore structures helped in maintaining a high specific capacitance at high C–D rates (high current density). By contrast, the CXs synthesized at high pH levels exhibited markedly low specific capacitances at low current densities. This finding was a consequence of their reduced specific surface areas and poorly developed pore structures.

During the synthesis of RF gels for CX production, pH played a crucial role in the initial nucleation. This nucleation process influenced the subsequent polymer growth and the formation of 3D network structures and pore structure development. Variations in the physical characteristics of CXs due to pH changes showed a direct correlation with the electrochemical performance of the EDLCs. Overall, a relatively low pH benefited particle growth, positively affecting the development of 3D network structures. Low pH conditions induced physical properties in the CXs, such as an increased specific surface area and an improved pore structure, which contributed to the high performance of the EDLCs. Thus, meticulous pH control during the polymerization process is crucial for optimizing the physical properties of CXs, thereby enhancing the overall efficiency and performance of EDLCs.

### 4.3. Ratio of Resorcinol to Catalyst

Figure 4c provides an overview of the mechanism of the CX structure underlying its influence on the molar R/C ratio. The R/C ratio was closely associated with the number of nuclei generated during the initial step of RF gel synthesis [41,57]. The nuclei formed during this initial step substantially influenced the physical properties of the CXs. Specifically, at a low R/C ratio, which indicates a high amount of catalyst, numerous nuclei formed, and the catalyst induced a vigorous reaction. Otherwise, at a high R/C ratio, which reflects a low amount of catalyst, a less vigorous reaction occurred due to the reduced catalyst amount.

**Figure 7:**
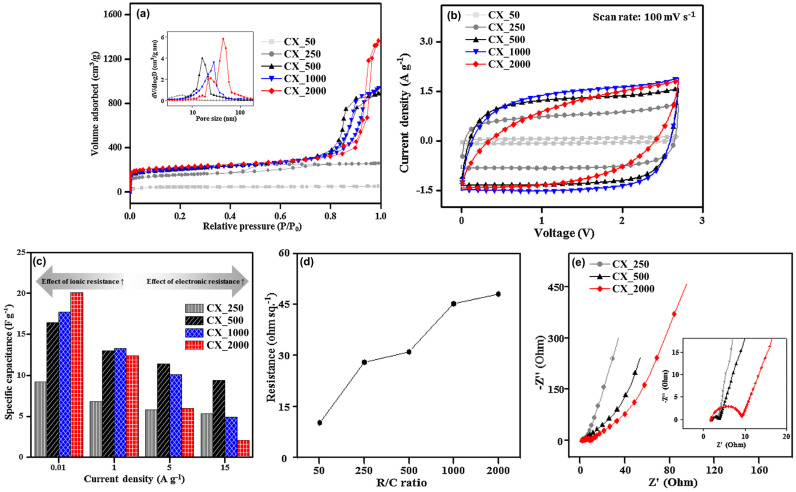
(**a**) N_2_ adsorption–desorption isotherm, (**b**) cyclic voltammograms at a scan rate of 100 mV/s, (**c**) specific capacitance to current density, (**d**) sheet resistance, and (**e**) Nyquist plots of carbon xerogels (CX_R/C ratio) [57].

**Figure 8:**
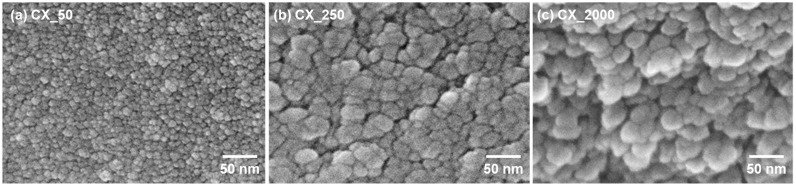
SEM image of carbon xerogels synthesized using different R/C ratios: (**a**) CX_50, (**b**) CX_250, and (**c**) CX_2000 [57].

As elucidated in Figure 7 and Table 5, an increase in the R/C ratio resulted in elevated specific surface areas, pore dimensions, and pore volumes. To facilitate a more intuitive understanding, scanning electron microscope (SEM) images of CXs according to the R/C ratio are displayed in Figure 8. As the R/C ratio increased, the proportion of catalyst per resorcinol decreased, resulting in fewer reaction initiation sites. The decrease in the number of reaction initiation sites implied the reduced formation of nuclei. This effect provided more space for particle growth, resulting in the formation of larger particles. Consequently, these larger particles formed a 3D network, leading to the development of relatively larger pores.

By contrast, CXs synthesized with a low R/C ratio suffered from limitations in pore structure development due to the large number of particles generated by the high catalyst quantity. Specifically, CX_50, which had a notably low R/C ratio, demonstrated a substantially reduced specific surface area and pore volume compared with other CXs. This finding suggests that excessively low R/C ratios resulted in the formation of particles that were too small to maintain a stable and effective 3D network structure of CXs for EDLCs.

Synthesized with a high R/C ratio, CX_2000 was expected to exhibit a superior electrochemical performance given its high specific surface area and abundant pores. However, the results from CV, GCD, and EIS analyses indicated unexpected deviations (Figure 7b, 7c, and 7e, respectively). Different from other CXs, CX_2000 displayed a rugby-ball-shaped CV curve at a scan rate of 100 mV/s, diverging from the expected rectangular shape. This finding suggests a decrease in the electrochemical efficiency. Furthermore, the GCD analysis highlighted that despite its notable specific capacitance at low current densities due to its high specific surface area, CX_2000 failed to maintain this capacitance at high current densities. In contrast, although CXs synthesized with a low R/C ratio exhibited a low specific capacitance at low current densities, they effectively maintained their specific capacitance at high current densities.

The electrochemical performance of a CX is intricately related to its physical and resistance properties. CXs fabricated at a high R/C ratio exhibited a high specific surface area and superior porosity. At a high R/C ratio, the development of large particles led to an improved pore structure but adversely affected the 3D network connectivity among particles. As a result, the reduced connectivity among particles increased the electronic resistance of the electrode material. Conversely, at a low R/C ratio, despite the less developed pore structure, leading to increased ionic resistance, the well-connected 3D network of particles considerably reduced the electronic resistance of the electrode material.

The structural modifications of the CXs substantially influenced the resistance properties of the EDLCs. This relationship was demonstrated by the analysis findings of the Nyquist plots and FPP measurements (Figure 7d,e). The Nyquist plots showed that the decrease in the R/C ratio led to a smaller semicircle size, which directly signified a low R_ct_ in the EDLC electrodes. The R_ct_ encompassed the ionic resistance, which involved the obstruction of electrolyte ion movement within the electrode materials, and the intrinsic electronic resistance of electrode materials themselves [58,145]. Despite the less developed pore structure of CXs fabricated with a low R/C ratio, which presents a disadvantage for ion transport, a low R_ct_ was observed. This result implies that the relatively high ionic resistance was compensated for by the low electronic resistance resulting from the well-connected 3D network structure. FPP measurements, which were used in the direct assessment of the electronic resistance of CXs, revealed that the electronic resistance increased with the increase in the R/C ratio. Thus, the CXs with a large pore size exhibited a high electronic resistance due to their poorly interconnected 3D carbon particle networks, which is consistent with and reinforces the conclusions drawn from other electrochemical analyses.

In conclusion, various studies have confirmed that the R/C ratio plays a crucial role in determining the physical properties and electrochemical performance of CXs [41,67]. Specifically, the importance of the R_ct_, which includes ionic and electronic resistances, for the electrochemical efficacy of EDLC cells should be emphasized. An exhaustive examination explored how the structural characteristics of CXs influence these resistance properties. In the synthesis of CXs for EDLC electrode materials, the R/C ratio emerged as a fundamental factor. This finding necessitates meticulous optimization to achieve an improved electrochemical performance of EDLCs.

## 5. ACXs for EDLCs

Activated carbons are used as electrode materials in commercial EDLCs because of their porous properties and extensive specific surface areas. These properties are advantageous as they provide a substantial area for electrolyte ion adsorption, which improves the capacitance of EDLCs. Therefore, CXs with large specific surface areas may exhibit superior electrochemical performances as electrode materials for EDLCs.

For the attainment of a high specific surface area in CXs, an additional activation process at high temperatures was conducted. This process produced ACXs using various acids, bases, or gases as activating agents [52,53,54,59,148]. The activation methods included chemical activation, which uses chemical activators (H_3_PO_4_, NaOH, and KOH), and physical activation, which employs gases (CO_2_ and steam). The preparation of commercial activated carbons for EDLCs mainly involves activation with steam or KOH, with KOH activation being particularly effective in increasing the specific surface area. Therefore, employing KOH activation in CXs to synthesize ACXs with a specific surface area and understanding the influence of physical property changes on electrochemical performance is essential.

**Figure 9:**
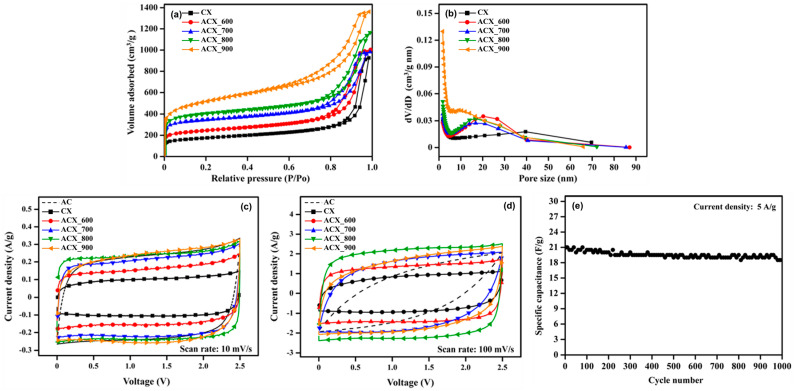
(**a**) N_2_ adsorption–desorption isotherm, (**b**) pore size distribution of activated carbon xerogels (ACX_activation temperature), (**c**) cyclic voltammograms at a scan rate of 10 mV/s and (**d**) 100 mV/s of activated carbon (AC) and activated carbon xerogels (ACX_activation temperature), and (**e**) cycle stability of ACX_800 [53].

During the activation reaction, micropores developed within the CXs, which led to an increase in the specific surface area. In addition, the activation reaction temperature contributed to determining the specific surface area and pore structure. Consequently, systematic research was conducted to explore the effect of the activation temperature on the electrochemical performance of ACXs [52,53]. Figure 9 illustrates the results of the physical and electrochemical analyses of ACXs. After the activation under various temperature conditions, the N_2_ adsorption–desorption isotherms exhibited hysteresis loops. This observation suggests that preservation of the 3D network structure and mesopores in the CXs occurred post-activation. Moreover, as the activation reaction progressed, the micropore development improved, increasing the specific surface area. Higher activation temperature was associated with an increase in the specific surface area and a decrease in the average pore size. This phenomenon was attributed to the vigorous progression of the reaction at high activation temperatures. Table 6 summarizes the specific surface areas and average pore sizes of the ACXs.

The increase in the specific surface area achieved through activation improved the adsorption space for electrolyte ions, which enhanced the capacitance across all C–D rates. The benefits of CX-based materials, such as the superior electrical conductivity provided by their 3D network structure and the abundance of pores, contributed to the improved performance of EDLCs. Notably, ACXs performed excellently under rapid C–D rates. As the activation temperature increased, capacitance showed an enhancement even at high C–D rates. However, a decline in capacitance was observed in the ACXs activated at excessively high temperatures (900 °C). Similarly, the CV curves for ACXs activated at high temperatures changed into a rugby-ball shape with the increase in scan rates. This finding suggests that the harsh activation temperatures caused the collapse of pores in the ACXs and 3D network structures, which resulted in elevated resistance and diminished capacitance. In conclusion, activation at an optimal temperature is necessary for the synthesis of ACXs for EDLC applications. Moreover, EDLCs that utilize ACXs as electrode materials effectively retained their initial capacitance after 1000 C–D cycles, which indicates their substantial potential for use in commercial EDLC products.

## 6. Cost-Efficient Production of CXs for EDLCs

**Figure 10:**
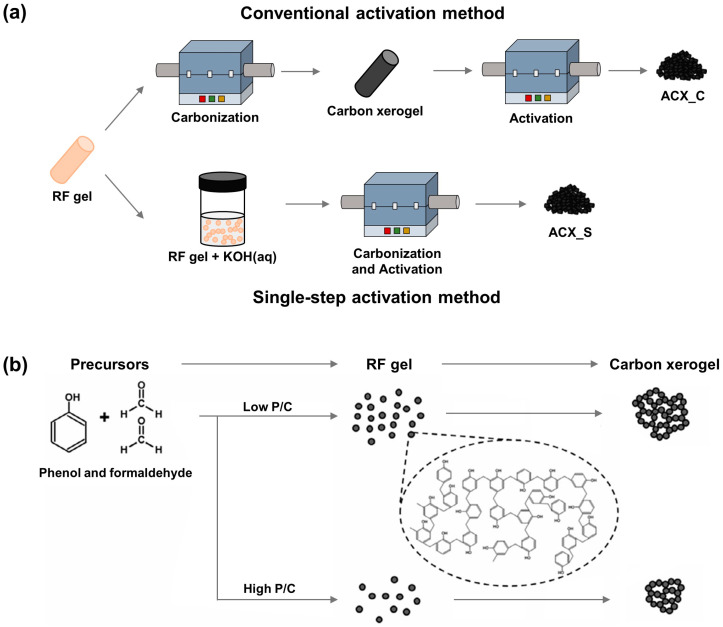
Preparation procedure for (**a**) activated carbon xerogels single-step activation method and (**b**) carbon xerogels using phenol precursor [54,60].

Various synthesis parameters and activation methods were investigated to synthesize CXs for EDLC electrode materials. Notably, ACXs exhibited superior electrochemical performance, which was attributed to their high specific surface area, abundant porosity, and 3D network structure. This observation underscores ACXs’ considerable potential as active materials for EDLC electrodes. Nevertheless, price competitiveness is crucial for the commercial success of ACXs, prompting research and development aimed at achieving cost-efficient production [149]. An understanding of the conversion mechanism of ACXs from their precursors has led us to propose two strategies for the cost-efficient manufacture of ACXs [54,60]. Figure 10 illustrates a schematic that aids in the comprehension of the proposed methods.

The first method involves a single-step activation process, which transforms RF gels directly into ACXs to provide an efficient solution [54]. CXs must be activated for the utilization of high-performance EDLCs. Additional procedures during the manufacturing stages contribute significantly to the reduced economic efficiency. The single-step activation process considerably expands the economic benefits by simplifying the process and thereby increasing the overall manufacturing efficiency. As a result, the single-step activation approach is considered a pivotal strategy for substantially boosting the commercial viability of ACXs.

The second method involves the use of phenol as a more cost-effective precursor compared to resorcinol [60]. Reducing the raw material costs directly affects the economic efficiency of the final products. Therefore, the utilization of inexpensive raw materials lowers the overall manufacturing costs and ultimately promotes the market competitiveness of the final product. Furthermore, phenol precursors may require unique manufacturing parameters that are distinct from those for resorcinol. One such parameter is the ratio of phenol to catalyst, which can lead to superior pore structures or improved electrochemical performance. Consequently, the application of phenol precursors is considered a critical strategy to improve the economic efficiency of the process and widen commercialization.

## 7. Comparison of CXs and Commercial Electrode Materials

**Figure 11:**
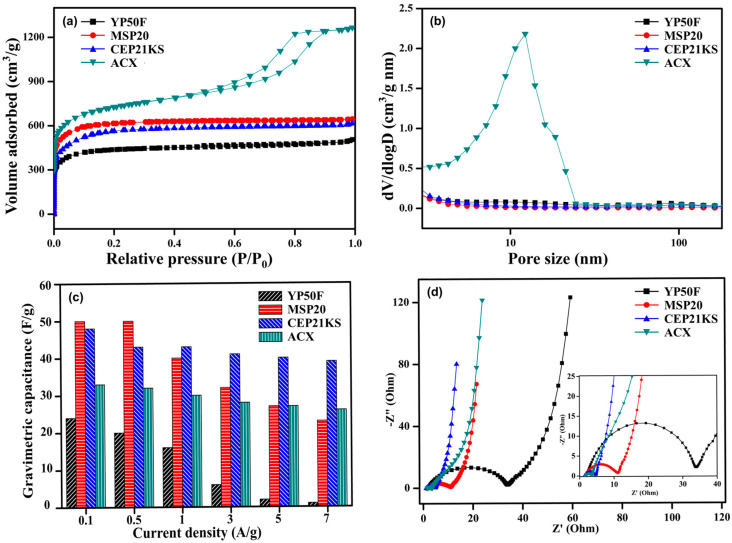
(**a**) N_2_ adsorption–desorption isotherm, (**b**) pore size distribution, (**c**) specific capacitance to current density, and (**d**) Nyquist plots of activated carbon materials [58].

Studies on the effect of structural modifications in CXs due to variations in the synthesis environment has shown the high potential of CXs as electrode materials for EDLCs. Note that rather than simply using CXs, utilizing ACXs can maximize the performance of EDLCs. Additionally, for practical applications, a comprehensive comparative analysis must be conducted between CXs and commercial activated carbon, which is commonly used as an electrode material for EDLCs, to ensure their applicability and effectiveness. Hence, using activation methods, we successfully synthesized an ACX with a superior performance and conducted a comparative analysis using commercial activated carbons (YP50f, MSP20, and CEP21KS) [58]. Figure 11 illustrates the physical attributes and electrochemical performance of this ACX compared with commercial activated carbons.

YP50F (Kuraray Co., Tokyo, Japan), MSP20 (Kansai Coke & Chemicals Co., Hyogo, Japan), and CEP21KS (Power Carbon Technology Co., Gumi, Republic of Korea) are high-quality activated carbons commercially available as electrode materials for EDLCs. Table 7 provides detailed properties of these commercial activated carbons, along with ACX. These carbon materials vary based on their precursor materials and activation method. YP50F, which is derived from coconut shells via physical activation, has a relatively low specific surface area and contains a high amount of impurities [150]. These properties suggest YP50F exhibits low capacitance due to its low specific surface area and high electrical resistance due to the presence of impurities. Empirical evaluations confirmed its inferior performance compared with other carbon materials. By contrast, MSP20 and CEP21KS, which were synthesized through chemical activation using KOH, substantially enhanced the electrochemical performance of EDLCs by providing a high specific surface area. As a result, MSP20 and CEP21KS demonstrated superior electrochemical properties compared with YP50F.

The ACX was synthesized via KOH activation and was characterized by its abundant micropores and mesopores, along with a 3D network structure that increased electrical conductivity. These properties provided the ACX with a high capacitance and low resistance, which ensured that its electrochemical performance was comparable to and potentially exceeded commercial standards. Notably, the ACX demonstrated exceptional capacitance retention under C–D rates, a feature closely related to its resistance properties. FPP measurements, which aim to determine the essential electronic resistance of electrode materials, showed that CEP21KS exhibited the lowest electronic resistance. Nonetheless, according to the Nyquist plots, the ACX possessed the smallest R_ct_, which suggests that the R_ct_ encompassed both electronic and ionic resistances. The low electronic resistance of the ACX was attributed to its highly interconnected 3D network structure, and the extensive porosity facilitated a low ionic resistance due to the abundance of ion pathways.

The ACX demonstrated considerable potential for improving the electrochemical performance of EDLCs, underscoring the importance of structural control and activation in optimizing their performance. In addition, we confirmed the industrial value of ACXs, which were specifically optimized for use as electrode materials of EDLCs.

## 8. Conclusions

This review comprehensively examined an array of research focused on the application of CXs, derived from resorcinol and formaldehyde, as electrode materials for EDLCs. It investigated the mechanism underlying the effects of adjusted synthesis parameters (such as the polymerization time, pH, and R/C ratio) on the structure of CXs. Detailed analyses of how these structural changes on the electrochemical performance of CXs were provided, highlighting the importance of appropriate synthesis conditions. Specifically, CXs comprising large particles exhibited low ionic resistance due to their large pore diameters and high pore volumes, whereas CXs made of smaller particles formed smaller pores but attained low electronic resistances through a highly developed 3D network. These observations emphasize the importance of precise synthesis conditions for CX preparation. The high specific surface area of electrode materials substantially contributes to the performance of EDLCs. This study explored how the use of an ACX, produced through the activation of CXs with KOH, can improve EDLC performance. The superior electrochemical performance of ACXs compared with CXs was elaborated and the influence of ACX activation temperature on EDLC performance was carefully examined. Through the design of the ACX structure for use as EDLC electrode materials and their comparison with commercial activated carbons (YP50F, MSP20, and CEP21KS), this review assessed the commercial viability of ACXs. ACXs exhibited a higher specific surface area and a more abundant pore structure than commercial activated carbons. Notably, ACXs exhibited the lowest charge transfer resistance (R_ct_), which is a critical factor directly associated with EDLC efficiency. The low R_ct_ of ACXs was attributed to their high porosity and superior 3D network connectivity, resulting in low electronic resistances. These results reveal the superior functionality of ACXs as electrode materials for EDLCs and underscore their extensive potential for commercial applications. In conclusion, tailoring the structure of ACXs is pivotal for the improved performance of EDLCs, with an emphasis on their potential as next-generation materials for efficient energy storage systems. The development and optimization of ACXs for EDLC applications were evaluated to accelerate the advancement of energy storage technology and open up new possibilities for technological innovation.

## Figures and Tables

**Table 1:** Characteristics of various carbon gels [62,65,66].

Type	Drying Method	Advantages	Disadvantages
Aerogel	Supercritical drying	The structure mostly remains intact after drying.	Aerogels require supercritical drying equipment, making them expensive to produce.
Xerogel	Ambient pressure drying	The manufacturing method is very simple and highly beneficial for mass production.	Xerogels experience more structural collapse than other gels.
Cryogel	Freeze drying	After drying, the structure remains partially intact.	Cryogels necessitate freeze-drying equipment and are costly to produce.

**Table 2:** Overview of EDLC electrode materials and their electrochemical performances.

Electrode Materials	Electrolyte	Specific Capacitance (F/g)	Current Density (A/g)	Reference
Activated carbon	6 M KOH in H_2_O	122	1.0	[106]
		262	0.05	[107]
		198	1.0	[108]
		148	0.5	[109]
		254	1.0	[110]
		93	0.1	[111]
		155	0.2	[112]
	1 M Na_2_SO_4_ in H_2_O	84	1.0	[113]
	1 M LiClO_4_ in PC	192	1.0	[114]
	1 M (C_2_H_5_)_4_NBF_4_ in PC	82	0.1	[115]
	1 M SBPBF_4_ in PC	82	1.0	[116]
	1 M TEABF_4_ in AN	115	0.2	[117]
	1 M TEABF_4_ in EC/DMC	88	1.0	[118]
	1 M TEABF_4_ in PC	41	1.0	[119]
	1 M Et_4_NBF_4_ in PC	299	1.0	[120]
	1 M Et_4_NBF_4_ in ACN	100	0.1	[121]
Carbon nanotube	1 M Na_2_SO_4_ in H_2_O	150	0.15	[122]
	1.96 M TEMABF_4_ in PC	44	2.0	[123]
	0.6 M TEABF4 in CPAME	96	0.5	[124]
Activated carbon fiber	6 M KOH in H_2_O	210	0.04	[125]
	1 M H_2_SO_4_ in H_2_O	248	1.0	[126]
	1 M TEABF_4_ in AN	112	1.0	[127]
Carbon aerogel	6 M KOH in H_2_O	384	0.01	[128]
		112	1.0	[129]
	1 M Et_4_NBF_4_ in AN	149	0.5	[130]
Carbon xerogel	6 M KOH in H_2_O	112	1.0	[45]
		201	1.0	[131]
		266	1.0	[132]
	1 M Na_2_SO_4_ in H_2_O	124	1.0	[133]
	1 M H_2_SO_4_ in H_2_O	141	0.2	[134]

**Table 3:** Physical properties of carbon xerogels (CX_polymerization duration) [55].

Samples	S_BET_ (m^2^/g) ^a^	D_avg_ (nm) ^b^	Total Pore Volume (cm^3^/g)
CX_1	645	12.2	0.7
CX_2	828	12.2	1.0
CX_5	796	16.1	1.2
CX_10	853	16.1	1.3
CX_20	998	21.3	2.1

^a^ Specific surface area was calculated by the Brunauer–Emmet–Teller (BET) plot. ^b^ Average pore diameter was determined by the Barrett–Joyner–Halenda (BJH) method with the desorption branch.

**Table 4:** Physical properties and specific capacitances (calculated from GCD measurements) of carbon xerogels (CX_pH) [56].

Samples	S_BET_ (m^2^/g) ^a^	D_avg_ (nm) ^b^	V_total_ (cm^3^/g) ^c^	Specific Capacitance (F/g)		
				1 A/g	3 A/g	5 A/g
CX_9.5	758.8	4.7	0.89	9.8	5.2	3.1
CX_10	613.0	2.9	0.45	8.0	4.4	3.2
CX_11	267.6	1.8	0.12	2.8	1.0	0.8
CX_12	36.8	3.1	0.03	0.9	-	-

^a^ Specific surface area was calculated by the BET plot. ^b^ Average pore diameter was determined by the BJH method with the desorption branch. ^c^ Total pore volume.

**Table 5:** Physical properties of carbon xerogels (CX_R/C ratio) [57].

Samples	S_BET_ (m^2^/g) ^a^	D_avg_ (nm) ^b^	Total Pore Volume (cm^3^/g)
CX_50	162.5	2.0	0.09
CX_250	564.4	2.8	0.43
CX_500	730.6	7.5	1.39
CX_1000	752.6	7.8	1.46
CX_2000	847.3	10.1	2.11

^a^ Specific surface area was calculated by the BET plot. ^b^ Average pore diameter was determined by the BJH method with the desorption branch.

**Table 6:** Physical properties and specific capacitances (calculated from GCD measurements) of carbon xerogel (CX) and activated carbon xerogels (ACX_activation temperature) [53].

Samples	S_BET_ (m^2^/g) ^a^	D_avg_ (nm) ^b^	Specific Capacitance (F/g)		
			1 A/g	3 A/g	5 A/g
CX	628	17.7	8.7	6.6	5.0
ACX_600	895	14.4	14.5	13.8	12.0
ACX_700	1291	12.3	20.7	16.8	14.0
ACX_800	1493	12.4	23.3	23.1	21.0
ACX_900	1860	7.53	21.2	16.5	14.0

^a^ Specific surface area was calculated by the BET plot. ^b^ Average pore diameter was determined by the BJH method with the desorption branch.

**Table 7:** Physical properties and specific capacitances (calculated from GCD measurements) of commercial activated carbons (YP50F, MSP20, and CEP21KS) and ACX [58].

	YP50F	MSP20	CEP21KS	ACX
Maker	Kuraray Co., Tokyo, Japan	Kansai Coke & Chemicals Co., Hyogo, Japan	Power Carbon Technology Co., Gumi, Republic of Korea	-
Activation method	Physical activation by steam	Chemical activation by KOH	Chemical activation by KOH	Chemical activation by KOH
Raw material	Coconut shell (hard carbon)	Phenolic resin (hard carbon)	Cokes (soft carbon)	Phenolic resin (hard carbon)
S_BET_ (m^2^/g) ^a^	1590	2330	2090	2690
D_avg_ (nm) ^b^	1.8	1.8	1.9	2.9
V_micro_ (cm^3^/g) ^c^	0.7	1.0	1.0	0.6
V_meso_ (cm^3^/g) ^d^	0.2	0.1	0.1	1.1
V_total_(cm^3^/g) ^e^	0.8	1.0	1.1	2.0
R_sheet_ (Ohm/sq.) ^f^	1504.0	442.9	281.0	377.8

^a^ Specific surface area was calculated by the BET plot. ^b^ Average pore diameter was determined by the BJH method with the desorption branch. ^c^ Micropore volume. ^d^ Mesopore volume. ^e^ Total pore volume. ^f^ Sheet resistance: confirmed by four-point probe measurement.

## Data Availability

Not applicable.
